# Modeling chronic pain interconnections using Bayesian networks: insights from the Qatar Biobank study

**DOI:** 10.3389/fpain.2025.1573465

**Published:** 2025-05-27

**Authors:** Aisha Ahmad M. A. Al-Khinji, Dhafer Malouche

**Affiliations:** ^1^ College of Medicine, Qatar University, Doha, Qatar; ^2^ Clinical Translational Science Research Group, QU Health, Qatar University, Doha, Qatar; ^3^ Department of Mathematics and Statistics, College of Arts and Sciences, Qatar University, Doha, Qatar

**Keywords:** Bayesian network, Qatar Biobank (QBB), pain interdependencies, conditional probabilities, systemic pain, probabilistic modeling

## Abstract

**Introduction:**

This study examines the interdependencies among different chronic pain locations and their relationships with age and gender, critical for effective clinical strategies.

**Methods:**

A Bayesian network approach was applied to 2,400 adult participants (18+ years; 50% male, 50% female) from the Qatar Biobank (QBB). Participants were categorized into young (18–35 years, 40.9%), middle-aged (36–60 years, 50.6%), and seniors (61+ years, 8.5%).

**Results:**

The model identified direct and indirect associations among pain locations and demographic factors, quantified by odds ratios (ORs). Younger females had the highest probability of headaches or migraines (48.6%) compared to younger males (31.2%), with probabilities decreasing across age (OR 1.917; 95% CI 1.609–2.284). Hand pain strongly correlated with hip pain (OR 8.691; 95% CI 6.074–12.434) and neck or shoulder pain (OR 4.451; 95% CI 3.302–6.000). Back pain was a key predictor of systemic pain, with a 37.9% probability of generalized pain when combined with hand pain (OR 7.682; 95% CI 5.293–11.149), dropping to 6.6% for back pain alone. Age, back pain, and foot pain collectively influenced knee pain, which reached 72.7% in older individuals with foot and back pain (OR 4.759; 95% CI 3.704–6.114).

**Discussion:**

These Bayesian network parameters explicitly reveal probabilistic interdependencies among pain locations, suggesting that targeted interventions for key anatomical regions could effectively mitigate broader chronic pain networks. The model also elucidates how demographic predispositions influence downstream pain patterns, providing a clear and actionable framework for personalized chronic pain management strategies.

## 1 Introduction

Chronic pain is a global health challenge, affecting over 30% of the population and significantly impacting quality of life, healthcare systems, and economies Cohen et al. (1). Unlike acute pain, chronic pain persists beyond its initial cause, often becoming a disease itself, necessitating a nuanced approach to diagnosing and managing pain that integrates biological, psychological, and social factors.

Innovative platforms like the Qatar Biobank (QBB) are pivotal in addressing regional health challenges. Established in 2012, QBB is a prospective, population-based cohort study exploring health trajectories among Qataris and long-term residents. By 2019, 17,065 participants (28% of the target population) were enrolled, contributing over 2 million biological samples and clinical data, forming a robust foundation for precision medicine Al Thani et al. (2).

Despite its recognized global impact—roughly 30% of adults experience chronic pain—its true overall burden in the Gulf Cooperation Council (GCC) and North Africa remains incompletely characterized. Condition-specific surveys report painful diabetic neuropathy in Saudi Arabia (3%–4%) Garoushi et al. (3), osteoarthritis in Niger (7%) Assadeck et al. (4), and diabetic neuropathy in Oman (2.5%–4.5%) Al-Zadjali et al. (5). However, a meta-analysis aggregating all musculoskeletal and systemic pain syndromes estimates that 30%–40% of Middle Eastern adults suffer chronic musculoskeletal pain—rates on par with global figures. Moreover, these regional studies have relied almost exclusively on descriptive analyses, leaving the interdependencies among pain sites and their demographic drivers unexplored. In particular, research applying Bayesian networks to uncover probabilistic dependencies across anatomical pain locations and demographic variables remains scarce in the GCC and North Africa.

**Figure 1:**
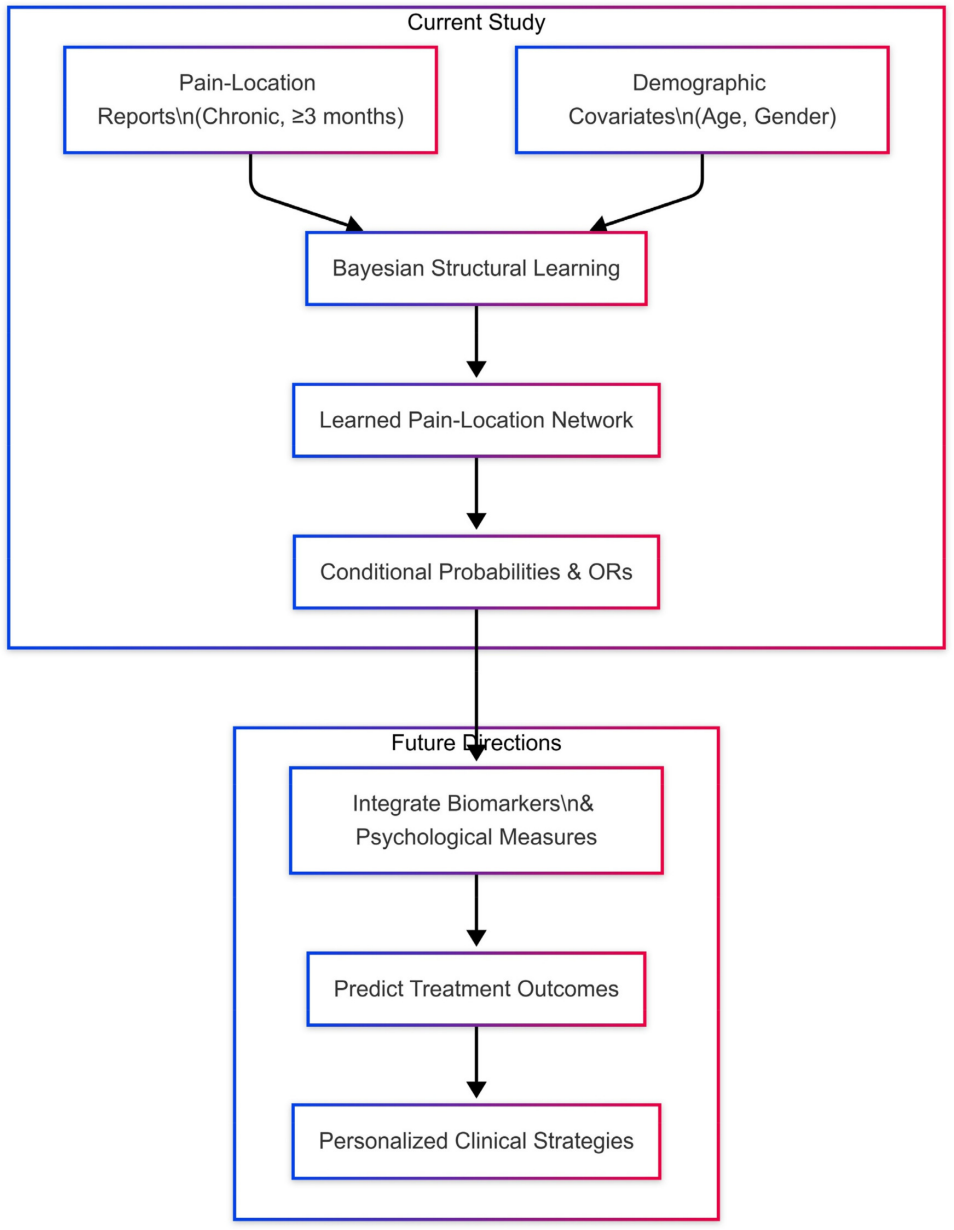
Conceptual overview of the study and its future extensions. In the Current Study, we combine self-reported chronic pain locations (*≥*3 months) with demographic covariates (age, gender) to perform Bayesian structural learning, yielding a directed pain-location network and associated conditional probabilities and odds ratios (ORs). In Future Directions, this foundational network will be augmented with biomarker and psychological data to predict treatment outcomes and guide personalized clinical strategies.

Traditional pain management approaches often fail to account for the complexity and interdependence of chronic pain locations. Bayesian Networks (BNs), by contrast, enable the modeling of conditional dependencies and directional relationships among multiple variables, making them well-suited for uncovering hidden structures within chronic pain data. This study addresses existing research gaps by applying Bayesian networks to model probabilistic and potential causal interconnections among chronic pain locations and demographic factors in Qatar. This modeling approach offers actionable insights to support targeted healthcare strategies, including early identification of individuals at risk for developing chronic pain, personalized pain management interventions, and resource allocation to effectively manage and prevent chronic pain in specific population groups. This preliminary exploration lays the groundwork for future integration of physiological, psychological, and treatment-response data (Figure 1). Such an approach aligns with recent advancements using Bayesian networks to model heterogeneity in populations, enabling personalized interventions based on demographic characteristics. Marchant et al. (6) recently showed that modeling a mixture of Bayesian networks—where the probability of each network component depends on individual covariates—uncovers distinct dependency structures across subpopulations and improves predictive accuracy. In the same spirit, our BN analysis of chronic pain demonstrates how demographic covariates (age, gender) not only influence marginal pain probabilities but also reshape the structure of inter-pain dependencies, thereby informing more personalized intervention strategies.

Bayesian Networks (BNs) are probabilistic graphical models that capture conditional dependencies among variable through a directed-acyclic graph, making them well suited to reveal how different chronic-pain sites interact across age and gender in our Qatar Biobank sample of binary pain indicators. Their transparent structure produces interpretable outputs—conditional probabilities, odds ratios, and easily visualized pathways—that can inform early, targeted interventions. Still, BN inferences are sensitive to data quality, variable selection, and the validity of assumed independencies; these factors must be considered when interpreting results derived from self-reported pain data and a relatively small senior subgroup.

Although previous studies have extensively described chronic pain prevalence and demographic associations, they often overlook the complex probabilistic interdependencies among pain locations. Utilizing a Bayesian network framework, this study explicitly identifies these interconnections, revealing how specific anatomical pain regions can influence broader pain networks. By mapping these relationships alongside demographic factors such as age and gender, our analysis offers a clear, actionable framework that can guide targeted diagnostic and therapeutic interventions, potentially disrupting chronic pain progression pathways at early stages.

## 2 Methods

### 2.1 Data collection

The dataset used in this study was sourced from the Qatar Biobank (QBB), a comprehensive resource established to advance research on health and disease within Qatar's population Al Thani et al. (2). It comprises data from 2,400 participants, evenly distributed by gender (50% female and 50% male), providing a balanced foundation for analysis. It's stratified by age: 40.9% young adults (18–35 years), 50.6% middle-aged (36–60 years), and 8.5% seniors (61+ years). Participants reported pain lasting more than three months via a structured question, with options including headache, back pain, and knee pain. Selecting “None of the above” precluded other choices, ensuring data integrity.

The dataset includes a diverse array of demographic and pain-related variables. Among these are 12 categorical variables representing distinct types of pain, which include headache or migraine, generalized body pain, facial pain, neck or shoulder pain, back pain, abdominal pain, hip pain, knee pain, hand pain, and foot pain.

Summary prevalence estimates for each pain location are provided in Section 3.1 (Table 1). These variations in pain prevalence underscore the importance of understanding the demographic and clinical contexts that may influence pain reporting.

**Table 1:** Summary of pain distribution across body regions with prevalence.

Body region	Pain present		Prevalence
	Yes	No	%
Headache	747	1,653	31.1
All Body	146	2,254	6.1
Face	69	2,331	2.9
Neck	824	1,576	34.3
Back	836	1,564	34.8
Stomach	523	1,877	21.8
Hip	160	2,240	6.7
Knee	597	1,803	24.9
Hand	214	2,186	8.9
Foot	301	2,099	12.5

Ethical considerations were rigorously maintained throughout the study. Data access and handling adhered to the ethical framework and regulations stipulated by the Qatar Biobank (QBB). The study protocol was approved by the Qatar Precision Health Institute Institutional Review Board (IRB) under project reference number QF-QBB-RES-ACC-00281, ensuring compliance with human subjects' research protections and confidentiality standards.

### 2.2 Analytical framework

The exploratory phase focused on conducting univariate analyses to understand the distributions of individual variables within the dataset. Bar plots were used extensively to summarize the prevalence and distribution of pain locations across various demographic categories. For instance, the prevalence of different pain locations was visualized by gender to highlight potential disparities, while age group distributions—classified as Young Adult, Middle-Aged, and Senior—were examined to identify demographic patterns in the data. Additionally, the gender distribution within age groups was analyzed to capture any notable trends. These visualizations, created using R software (version 4.4.2) and the *ggplot2* and *sjPlot* packages, provided a clear and comprehensive understanding of the dataset, serving as a foundation for subsequent analyses, including the development of Bayesian networks.

### 2.3 Bayesian network development

To model the probabilistic relationships between pain locations and demographic factors, we applied a Bayesian Network (BN) methodology. Bayesian Networks are represented as Directed Acyclic Graphs (DAGs), where arrows indicate conditional dependencies between variables. The structure of the network was learned using a score-based approach, specifically the Hill-Climbing (HC) algorithm, with the Bayesian Information Criterion (BIC) as the scoring function Gámez et al. (7). To maintain logical and medical consistency, we introduced constraints ensuring that demographic variables (age and gender) do not receive arrows from other variables, nor do arrows exist between these two demographic variables. This prevents any interpretation suggesting that factors like pain could alter a participant's age or gender, which is neither logically nor medically valid.

Following the structure learning phase, Maximum Likelihood Estimation (MLE) was utilized to estimate the parameters of the Bayesian Network. This process produced Conditional Probability Tables (CPTs) for each node based on its parent nodes, enabling a detailed quantification of probabilistic relationships. To further explore the relationships identified in the Bayesian Network, we calculated Odds Ratios (ORs) and their 95% Confidence Intervals (CIs). The Odds Ratio was defined as:

(1) $$OR=\;\frac{P\;\;(\mathrm{Event}|\mathrm{Evidence})\;}{P\;\;(\mathrm{No}\;\;\mathrm{Event}|\mathrm{Evidence})}÷\frac{P\;\;(\mathrm{Event}|No\;\mathrm{Evidence})\;}{P\;\;(\mathrm{No}\;\mathrm{Event}|No\;\mathrm{Evidence})}\;$$

where P(Event|Evidence) represents the probability of the event (e.g., back pain) occurring given a specific evidence (e.g., age group: senior). The 95% CI for the OR was calculated using the formula for the standard error of the ln(OR):

(2) $$SE_{\ln\;(OR)}=\sqrt{\frac{1}{a}+\frac{1}{b}+\frac{1}{c}+\frac{1}{d}\;}$$

where a,b,c,d are the counts in a 2 *×* 2 contingency table. Specifically:

• a: Number of events with the evidence present,
• b: Number of no events with the evidence present,
• c: Number of events with the evidence absent,
• d: Number of no events with the evidence absent.

The confidence interval was then derived as:

(3) $$95\text{\%}\;\;CI=\exp\;(\ln(OR\pm 1.96\;\times \;SE_{\ln\;(OR)}))$$

Equations (1–3) together provide the basis for quantifying and interpreting the probabilistic relationships revealed by the Bayesian Network.

This approach allowed a comprehensive evaluation of the strength and direction of associations both among different pain locations and between these pain locations and demographic variables. The analysis, visualization, and computation of Odds Ratios (ORs) and their Confidence Intervals (CIs) were performed using the bnlearn package (version 5.01) in R. Log-scale plots of ORs with CIs were generated to visually convey the magnitude and uncertainty of these associations, while graphical representations of the Bayesian Network highlighted the probabilistic dependencies among variables. This comprehensive approach provided a nuanced understanding of the interrelationships between pain locations and demographic factors. Furthermore, the methodology and findings are consistent with established research in Bayesian modeling applied to pain analysis Eckert et al. (8); Gamez et al. (7); Arias et al. (9), reinforcing the validity and relevance of this framework for exploring complex data relationships.

Our findings align broadly with existing Bayesian modeling approaches applied in chronic pain research Eckert et al. (8), highlighting the potential of Bayesian methods to identify probabilistic and conditional relationships among pain variables. While Eckert et al. utilized more complex physiological variables, our analysis demonstrates that even basic demographic and clinical variables (sex, age, pain locations) can yield valuable predictive insights. Additionally, the methodological framework of structural Bayesian network learning employed here follows established hill-climbing algorithms, previously validated for balancing efficiency and accuracy in general structural learning contexts Gamez et al. (7); Arias et al. (9).

## 3 Results

### 3.1 Descriptive statistics

The study includes 2,400 participants selected by sex-stratified sampling, resulting in 1,200 females and 1,200 males. Table 1 lists pain-location prevalences lasting ¿3 months: back (34.8%), neck (34.3%), headache (31.1%), knee (24.9%), stomach (21.8%), foot (12.5%), hip (6.7%), all-body (6.1%), and facial (2.9%).

**Figure 2:**
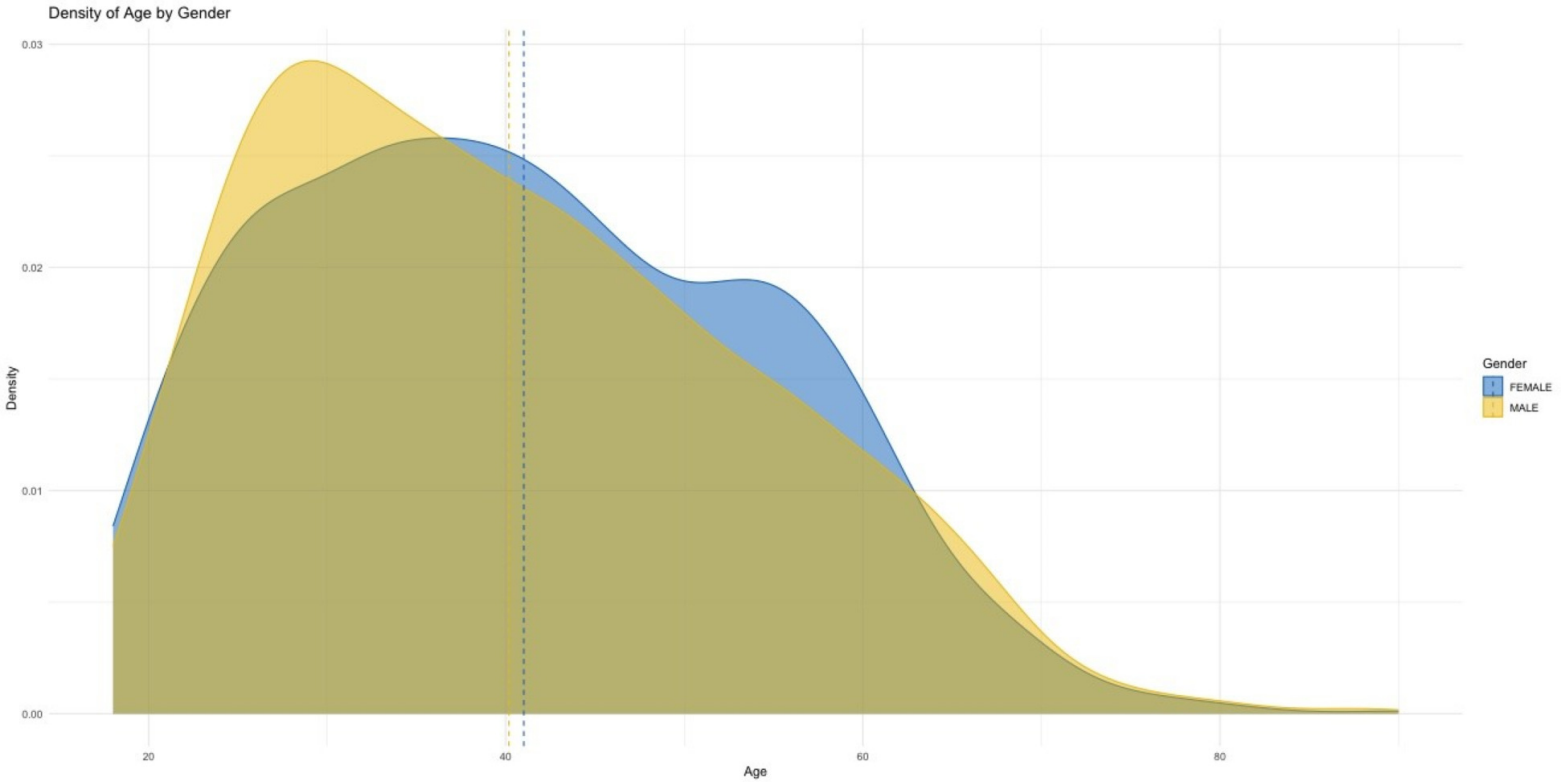
Age distribution by gender: blue for females, yellow for males, with dashed lines marking mean ages. Both genders show similar distributions, concentrated between ages 20 and 60.

Figure 2 shows that females report most pain sites more often than males, especially hand pain (70.1% vs. 29.9%) and generalized body pain (71.9% vs. 28.1%). These disparities may relate to hormonal, autoimmune, or stress-linked factors.

Age densities indicate slightly more young males (30–40 years) and a marginally higher male mean age. Both tails (<20 and >60 years) are thin; seniors (≥61 years) represent only 8.5% of the sample, limiting inferences for older adults.

**Figure 3:**
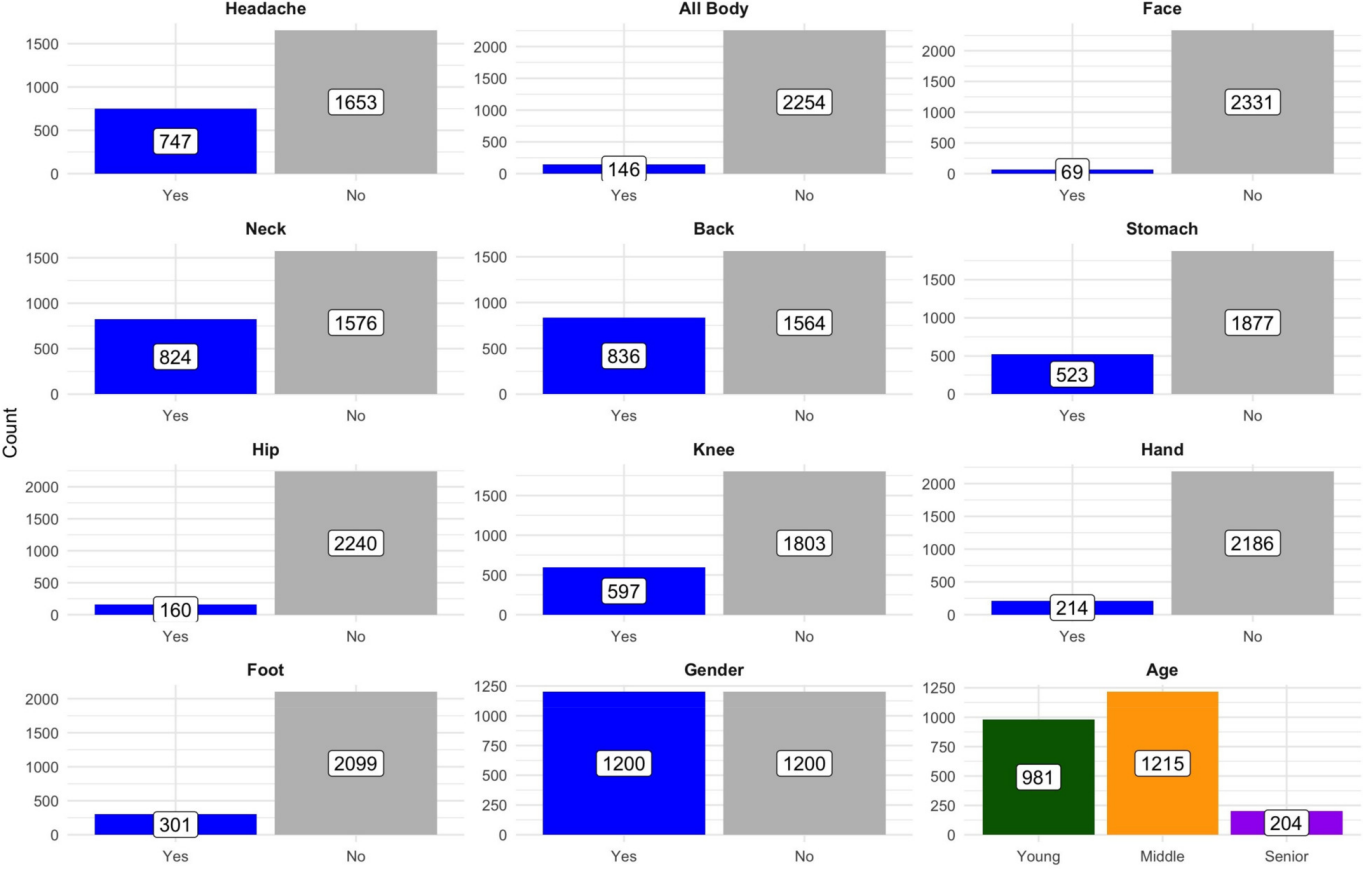
Distribution of counts across categories by variable: the plot shows the breakdown of counts for various health-related categories (e.g., headache, neck pain) and demographics (e.g., gender, age groups) across subcategories. Data are presented in facets for clarity, with counts annotated on the bars.

Figure 3 complements Table 1, visualizing the distribution across body regions. The dominance of back and neck pain, contrasted with rarer facial and hip pain, underlines heterogeneous burden and sets the context for subsequent network analyses linking pain locations to demographics.

### 3.2 Pain location by gender

**Figure 4:**
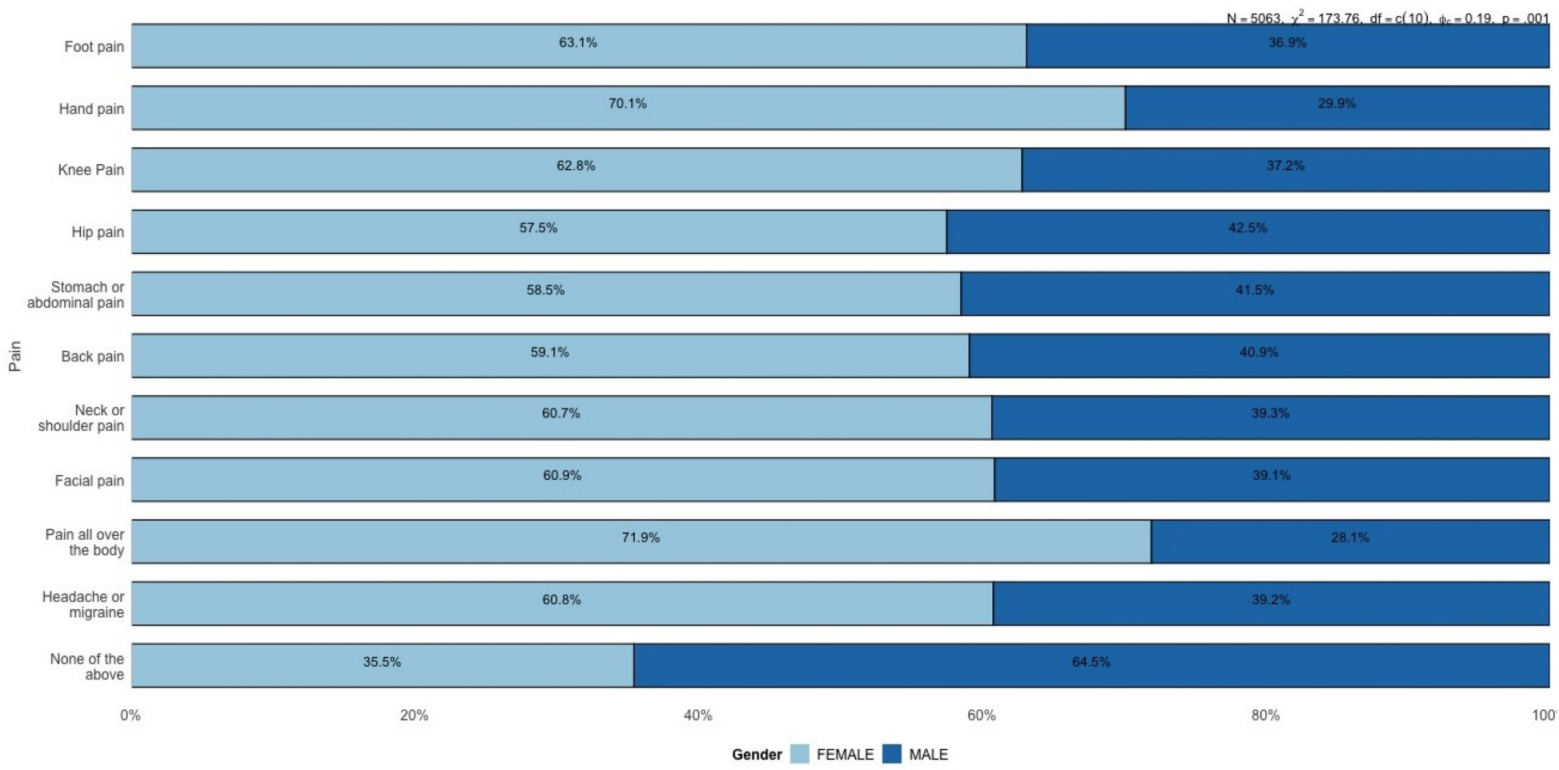
Proportion of reported pain locations by gender.

Figure 4 compares the prevalence of various pain locations between males and females. Females consistently report higher proportions for most pain locations, notably hand pain (70.1% females vs. 29.9% males) and generalized body pain (71.9% females vs. 28.1% males).

Knee pain prevalence was 62.8% among females vs. 37.2% among males. Hip pain was reported by 57.5% of females and 42.5% of males. Finally, 64.5% of males and 35.5% of females selected “None of the above” (no chronic pain) on the survey. This result could reflect gender differences in lifestyle, occupational hazards, physical activity, or injury-related factors that disproportionately affect men. Additionally, males are significantly more likely to report experiencing no pain (categorized as “None of the above”), with 64.5% of males reporting no significant pain compared to only 35.5% of females. This finding suggests that males generally experience a lower prevalence of reported pain or may underreport pain due to cultural or psychological factors related to pain perception and reporting.

**Figure 5:**
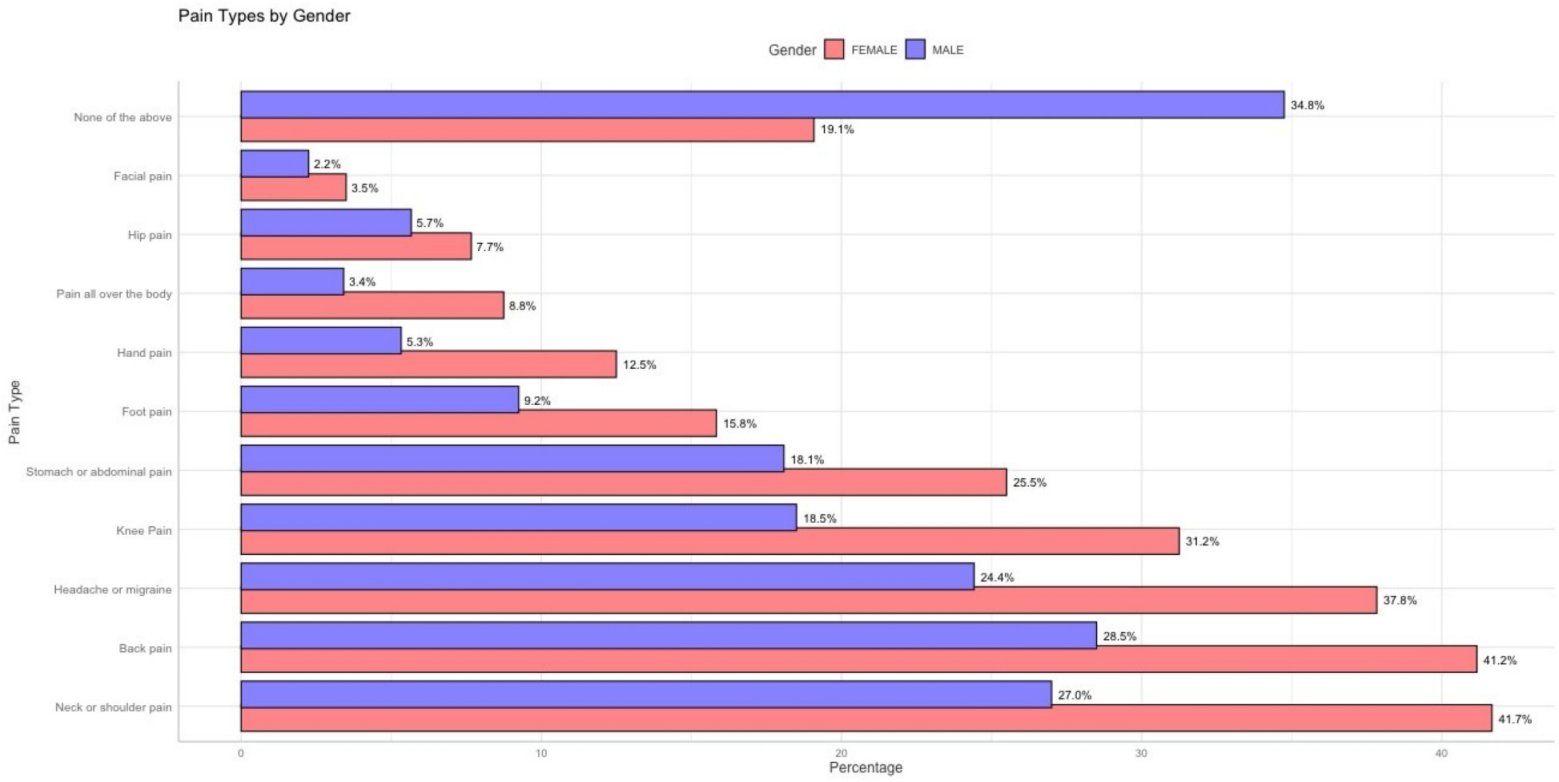
Percentage distribution of reported pain locations by gender.

Figure 5 further emphasizes the gender differences observed in specific pain locations, particularly facial pain and stomach or abdominal pain. Females report a higher prevalence of facial pain (3.5%) compared to males (2.2%). While the overall proportions of facial pain remain low in both groups, the difference is still noteworthy. Facial pain can often be associated with conditions like migraines, temporomandibular disorders (TMD), or sinus-related issues, all of which are more commonly reported in females. This disparity may highlight underlying physiological, hormonal, or stress-related factors that disproportionately affect women. In contrast, some studies suggest that males may underreport pain due to sociocultural norms and gendered expectations around stoicism and emotional expression, which could contribute to lower observed rates of pain reporting in men Wade et al. (10); Keogh (11).

A more substantial difference is observed in stomach or abdominal pain, where the prevalence in females reaches 25.5% compared to 18.1% in males. The higher proportion in females may be related to conditions such as irritable bowel syndrome (IBS), menstrual pain, or other gastrointestinal issues, all of which are more prevalent among women. Hormonal changes, particularly during the menstrual cycle or menopause, could further contribute to these differences, underscoring the need for gender-specific clinical attention to abdominal and gastrointestinal complaints.

These findings, in conjunction with Figure 4, reinforce the overall trend that females experience and report pain across more body regions compared to males. While descriptive statistics highlight these disparities effectively, they remain limited in understanding the conditional dependencies between different types of pain. For example, it is unclear whether females reporting stomach pain are more likely to experience generalized pain or whether facial pain is linked to headaches or migraines.

### 3.3 Pain by age

**Figure 6:**
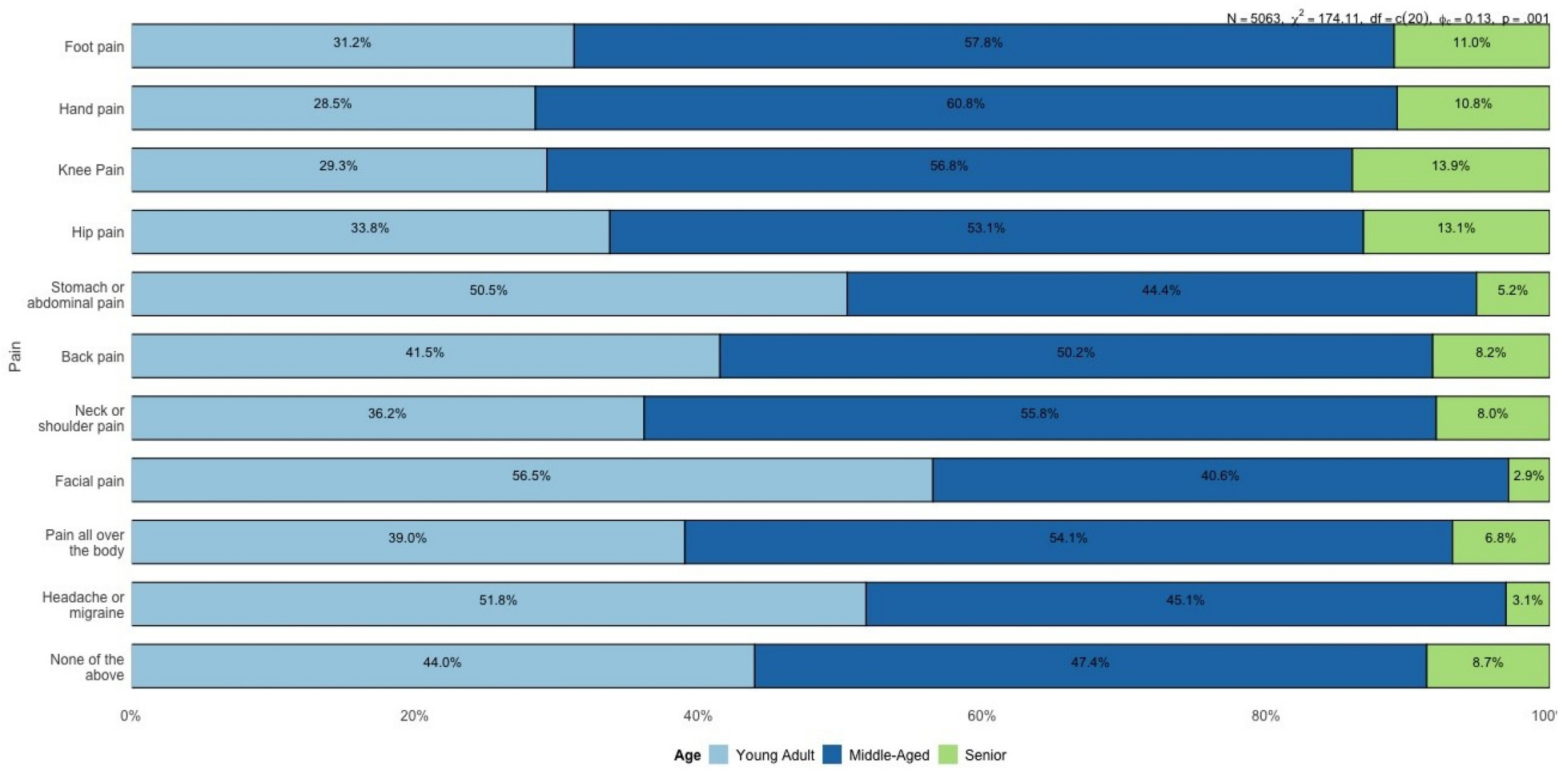
Proportion of reported pain locations by age group (young adults, middle-aged, and seniors).

Figure 6 highlights the variation in pain prevalence across different age categories. Young adults report the highest prevalence of headaches or migraines (51.8%) and stomach or abdominal pain (50.5%). These findings suggest a greater burden of systemic and stress-related conditions in this age group. Headaches and migraines are often associated with lifestyle stress, hormonal influences, and increased exposure to screen time or irregular sleep patterns Alotaibi (12); Seng et al. (13). Similarly, stomach or abdominal pain in younger adults could be linked to conditions like irritable bowel syndrome (IBS), dietary habits, or stress-induced gastrointestinal issues, all of which are common in younger populations.

**Figure 7:**
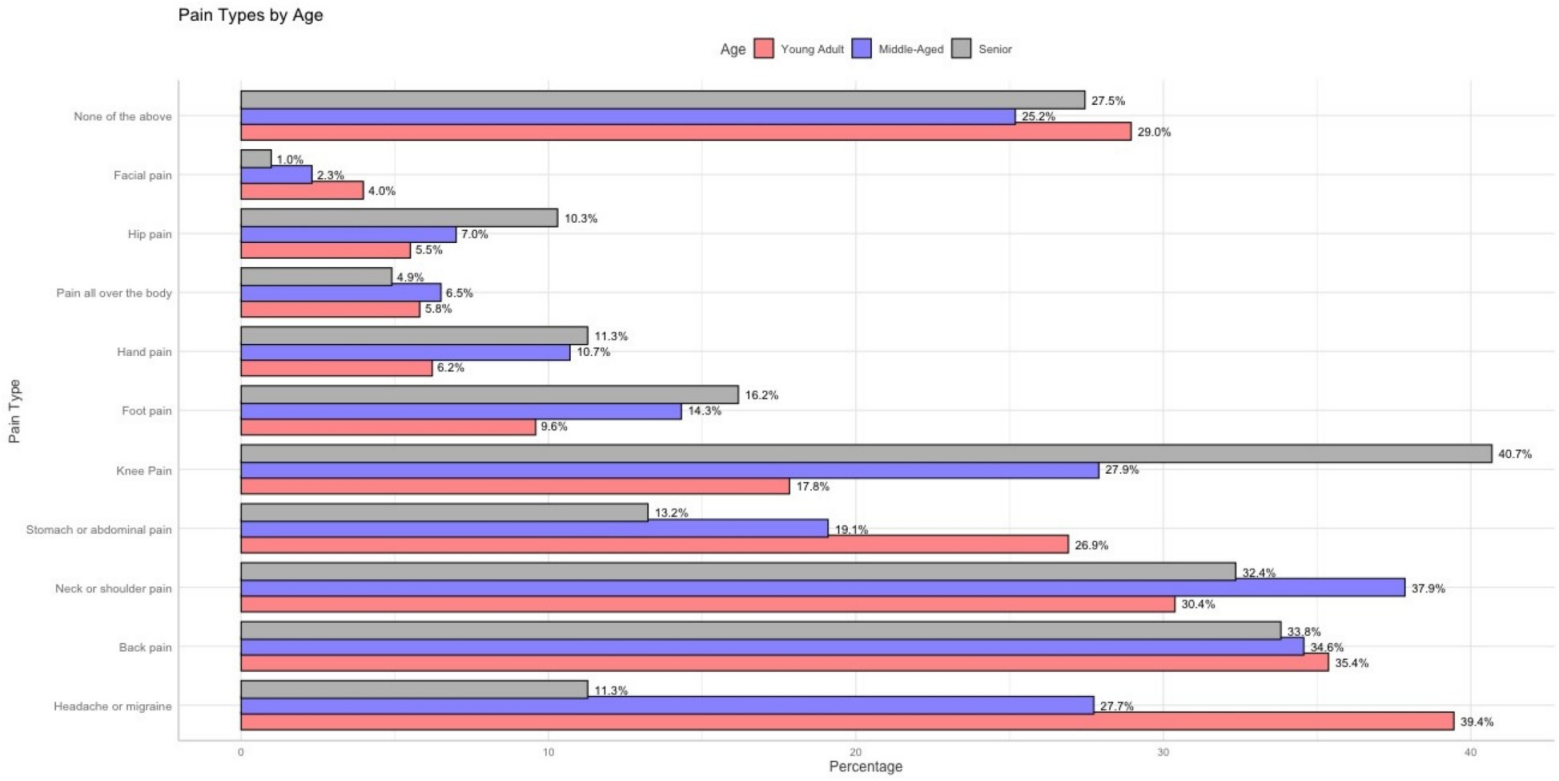
Percentage distribution of reported pain locations by age group (young adults, middle-aged, and seniors).

An interesting trend is observed in the “None of the above” category (see Figure 7), where 27.5% of seniors report no significant pain, compared to 25.2% of middle-aged individuals and 29.0% of younger adults. This could suggest that while seniors experience more localized and musculoskeletal pain, the absence of systemic pain locations might contribute to an overall perception of better pain management.

The progression of pain locations across age groups reveals a clear trend: systemic pain (e.g., headaches, stomach pain) dominates in younger adults, while localized musculoskeletal pain (e.g., back pain, knee pain, and foot pain) becomes increasingly prevalent in middle-aged and senior populations. This transition aligns with known patterns of age-related degeneration, changes in physical activity, and lifestyle factors. The descriptive analysis of pain locations across gender and age groups reveals important trends, such as the higher prevalence of systemic pain (e.g., headaches and abdominal pain) among younger adults and females, and the increased occurrence of localized musculoskeletal pain (e.g., back, knee, and hip pain) in middle-aged and senior populations. However, these observations remain isolated and fail to uncover the underlying conditional relationships between different types of pain. For instance, it is unclear whether hand pain influences the likelihood of back pain, or if neck pain acts as a precursor to systemic pain such as generalized body pain. Similarly, the role of demographic variables like gender and age in mediating these dependencies cannot be fully explained through descriptive statistics alone.

To address these gaps, a Bayesian Network was constructed to model the probabilistic relationships between pain locations and demographic factors. This network provides a graphical representation of conditional dependencies, allowing us to identify the pathways through which certain pains are connected and influenced by demographic variables. The following section presents the structure and findings of the Bayesian Network, highlighting key relationships and their clinical implications.

### 3.4 Probabilistic analysis of pain patterns with a Bayesian network

**Figure 8:**
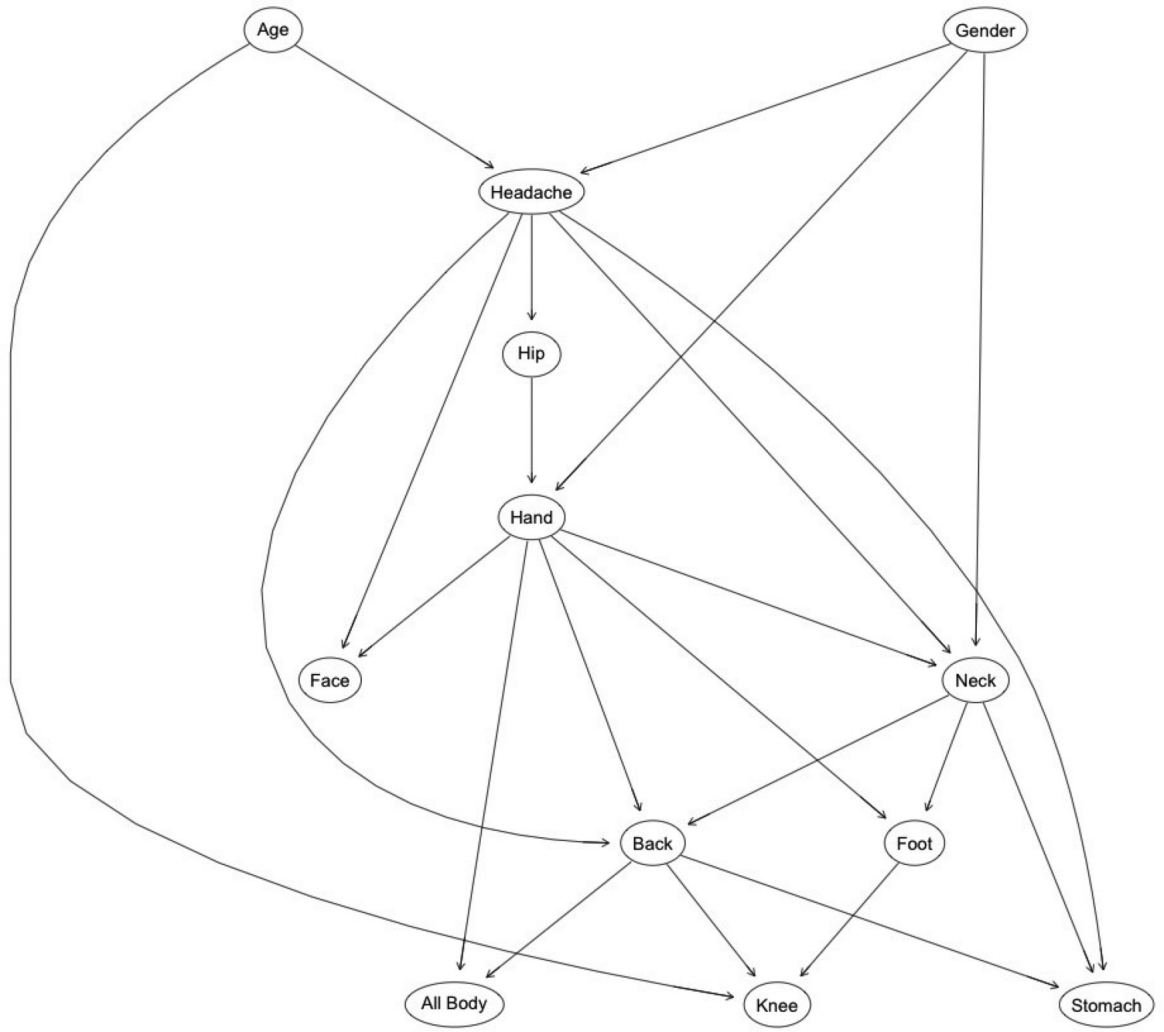
Bayesian network structure illustrating conditional dependencies among pain locations, age, and gender. Arrows represent the direction of conditional dependence; specifically, an arrow from variable A to variable B indicates that B is conditionally dependent on A. This structure captures the probabilistic relationships identified in the dataset using a score-based structure learning algorithm.

**Figure 9:**
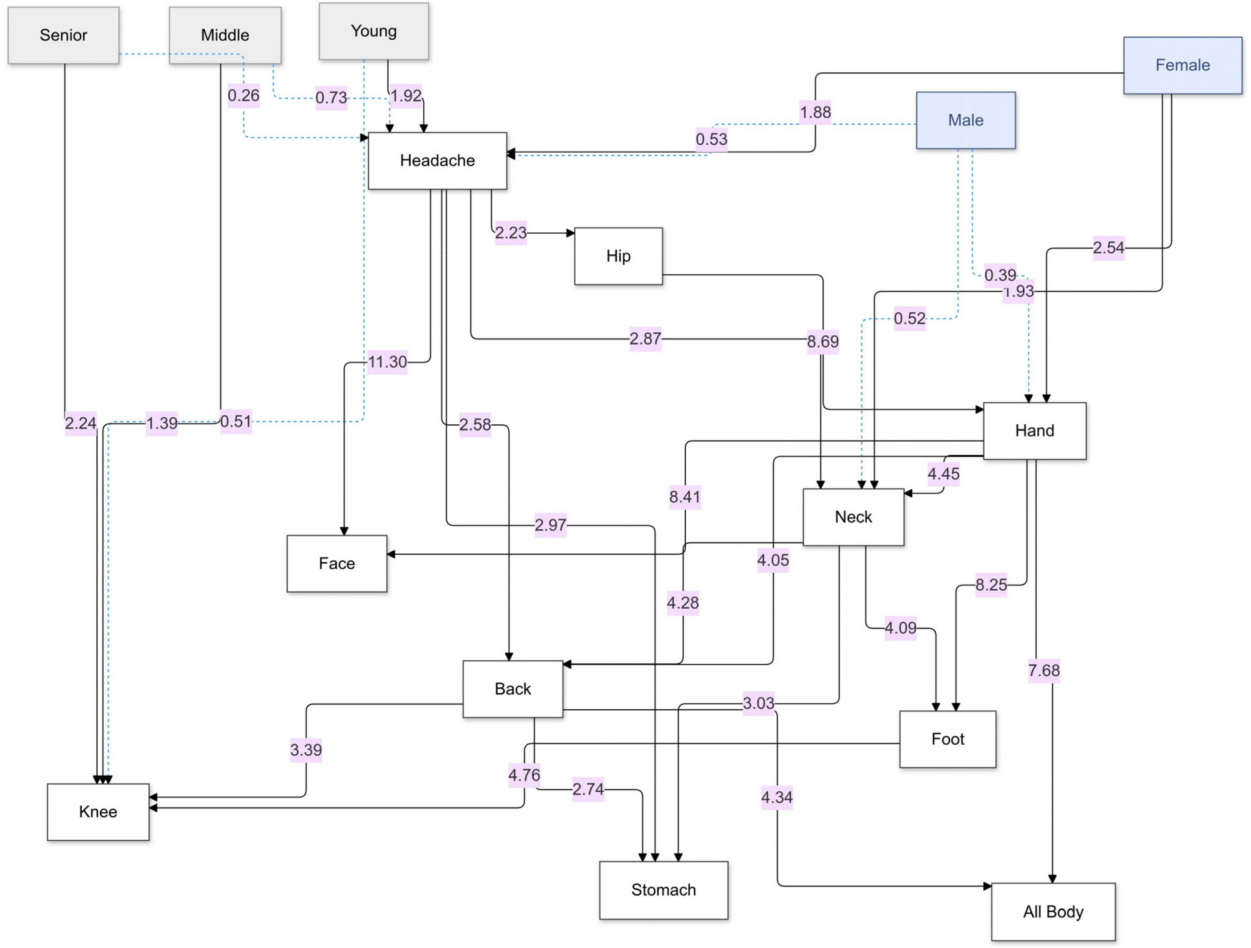
Bayesian network of pain relationships: this graph shows probabilistic links between age group, gender, and reported pain in various body regions, highlighting directional dependencies and associated odds ratios (ORs). Arrows represent the direction of conditional dependence; an arrow from variable A to variable B indicates that B is conditionally dependent on A. Solid black arrows denote statistically significant relationships (OR *>* 1 with 95% CI not crossing 1), while dotted blue arrows represent inverse or non-significant associations. The numerical values adjacent to the arrows are the corresponding ORs derived from the conditional probability Table 2.

To understand the probabilistic relationships among age, gender, and pain occurrence in various body regions, we employed a Bayesian Network (BN) analysis. The BN provides an intuitive graphical representation of these associations, supported by Odds Ratios (ORs) to quantify the strength of each relationship. Two versions of the BN were developed: one displaying the structural connections without ORs (Figure 8) and another enriched with annotated OR values (Figure 9).

Figure 8 offers a structural overview of the dependencies among age, gender, and pain occurrence across different body regions. The network reveals directional relationships, such as the pathways from demographic factors (e.g., “Age” and “Gender”) to specific pain locations (e.g., “Headache” and “Knee pain”), and subsequent connections between pain regions (e.g., “Headache” influencing “Neck” and “Back”). These pathways highlight the cascading effects of demographic factors on the propagation of pain through interconnected regions. For example, “Age” and “Gender” not only directly influence primary pain locations but also indirectly affect secondary regions such as the “Back” and “Stomach” through intermediary nodes like “Headache” or “Neck”. This structure underscores the probabilistic interdependence of pain locations, suggesting that interventions targeting specific regions may mitigate broader discomfort networks. By mapping these relationships, the network provides a clear framework for understanding how demographic predispositions set the stage for downstream symptom patterns, forming a cohesive and actionable probabilistic framework.

Figure 9 provides a detailed visualization of structural relationships, emphasizing the strength of associations between various pain locations as represented by annotated odds ratios (ORs). These ORs quantitatively measure the likelihood of co-occurrence between conditions, offering insights into key patterns.

For instance, headaches emerge as a pivotal node in the pain network. The odds ratio (OR) of 2.87 linking headaches to neck pain underscores a nearly threefold increase in the likelihood of co-occurrence. Similarly, the associations with hip pain (OR = 2.23) and stomach pain (OR = 2.97) highlight the systemic nature of headache impact. The calculation of OR = 2.87 is based on the probabilities:

$$\begin{array}{rl} & P\;(\mathrm{Neck}\;\mathrm{Pain}\;|\;\mathrm{Headache}\;\;\mathrm{Pain})=0.51138\;\mathrm{and}\\ & P\;(\mathrm{Neck}\;\;\mathrm{Pain}\;|\;\mathrm{No}\;\mathrm{Headache}\;\mathrm{Pain}=\;0.26739 \end{array}$$

(from Table 2), using the standard formula for odds ratios (see Equation 1).

$$OR=\frac{0.51138}{1-0.51138}÷\frac{0.26739}{1-0.26739}\approx 2.87$$

This precise computation highlights the strong relationship between headaches and neck pain as observed in the data.

**Table 2:** Summary of probabilistic relationships, their corresponding percentages of “Yes” and “No” outcomes, odds ratios (OR), and 95% confidence intervals (CI) derived from the Bayesian network analysis.

Relationship	Yes (%)	No (%)	Odds Ratio	95% CI	
				Lower	Upper
Age:Young → Headache	39.450	25.370	1.917	1.609	2.284
Age:Middle → Headache	27.737	34.599	0.726	0.610	0.863
Age:Senior → Headache	11.275	32.969	0.258	0.166	0.402
Age:Young → Knee	17.839	29.739	0.513	0.420	0.626
Age:Middle → Knee	27.901	21.772	1.390	1.154	1.675
Age:Senior → Knee	40.686	23.406	2.245	1.669	3.019
Gender:Female → Headache	37.833	24.417	1.884	1.580	2.246
Gender:Female → Hand	12.500	5.333	2.536	1.870	3.438
Gender:Female → Neck	41.667	27.000	1.931	1.627	2.293
Headache → Neck	51.138	26.739	2.867	2.395	3.433
Headache → Hip	10.442	4.961	2.234	1.617	3.085
Headache → Stomach	35.475	15.608	2.973	2.433	3.632
Headache → Face	7.631	0.726	11.297	6.024	21.186
Headache → Back	50.067	27.949	2.585	2.161	3.092
Hip → Hand	38.750	6.786	8.691	6.074	12.434
Hand → Face	13.551	1.830	8.410	5.096	13.880
Hand → Foot	45.794	9.286	8.253	6.082	11.199
Hand → All Body	25.234	4.209	7.682	5.293	11.149
Hand → Back	65.421	31.839	4.050	3.013	5.444
Hand → Neck	66.822	31.153	4.451	3.302	6.000
Neck → Back	56.675	23.414	4.279	3.572	5.125
Neck → Foot	23.301	6.916	4.089	3.175	5.265
Neck → Stomach	34.830	14.975	3.035	2.486	3.704
Back → Stomach	33.493	15.537	2.738	2.245	3.338
Back → Knee	40.311	16.624	3.387	2.796	4.104
Back → All Body	11.842	3.005	4.336	3.030	6.203
Foot → Knee	55.150	20.534	4.759	3.704	6.114

Table 2 provides detailed demographic probabilities highlighting meaningful age and gender differences in headache, hand, and neck pain.

Gender-based differences also show pronounced disparities. Among females, 37.83% report headaches, a stark contrast to the 24.42% in males, reflecting their greater predisposition. Similarly, females demonstrate elevated probabilities of reporting hand pain (11.95%) compared to males (5.16%). This pattern is echoed in neck pain, where 29.47% of females report the condition, compared to 16.92% of males, suggesting nearly one-third of females are affected.

Inter-symptom relationships further clarify the interconnected nature of pain locations. For example, among those with headaches, 50.067% also report back pain, compared to 27.95% of those without headaches. This suggests that more than half of headache sufferers experience back pain. Similarly, 28.03% of individuals with headaches report stomach pain, compared to only 10.78% of those without. This indicates that over one-quarter of headache sufferers are burdened with stomach pain, pointing to systemic implications. Facial pain is particularly striking, with 5.65% of individuals with headaches reporting it, a stark contrast to just 0.52% of those without headaches.

Finally, pain in specific regions reveals predictive patterns. Among individuals with hip pain, 19.09% report hand pain, far exceeding the 2.38% seen in those without hip pain. Similarly, hand pain is reported by 28.65% of individuals with foot pain, compared to only 4.22% in those without. This demonstrates that over one-quarter of individuals with foot pain concurrently experience hand pain, emphasizing the interconnectedness of pain locations across regions. These probabilities underscore the pervasive and overlapping nature of pain syndromes, reinforcing the necessity of targeted interventions tailored to specific demographic and symptom profiles.

**Figure 10:**
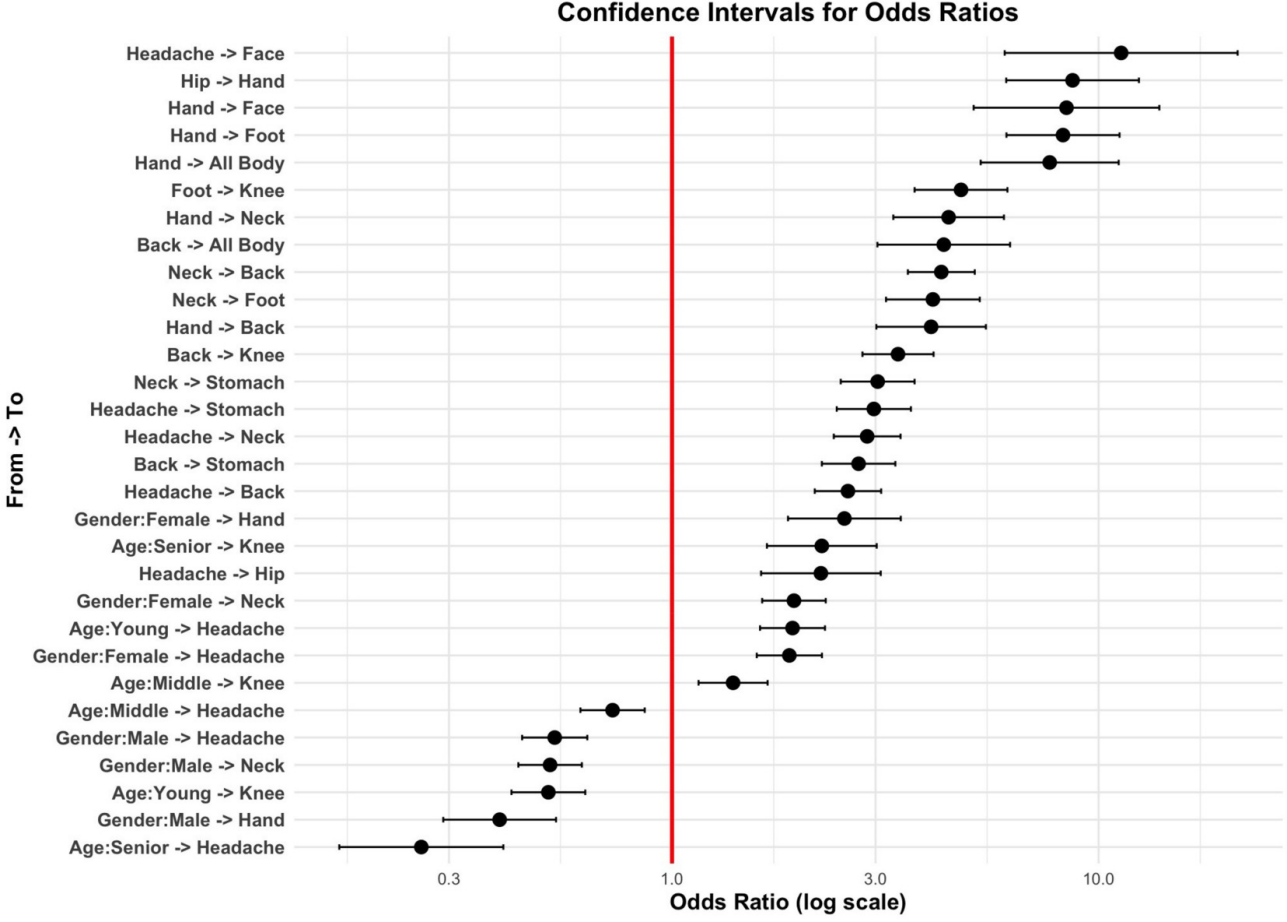
Confidence intervals for odds ratios (OR) of pain relationships, showing significant positive (right of OR = 1) and negative (left of OR = 1) associations, ordered by OR magnitude on a log scale.

Figure 10 illustrates the odds ratios (ORs) and 95% confidence intervals (CIs) for various demographic and inter-symptom relationships related to pain locations. It highlights notable patterns, such as the higher likelihood of younger individuals experiencing headaches (OR > 1), while middle-aged and senior groups have lower odds, reflecting an age-related protective trend. Gender-based disparities are evident, with females showing increased odds for headaches, neck pain, and hand pain compared to males. Strong inter-symptom connections emerge, such as the significant association between headaches and back pain, stomach pain, and facial pain, with consistently elevated ORs. Similarly, hip pain is closely linked to hand pain, while foot pain is strongly associated with knee and hand pain. ORs above 1 (with CIs not crossing 1) confirm these positive associations, while ORs below 1 reveal inverse relationships, such as the reduced likelihood of headaches in seniors. This graph underscores the interconnected nature of pain locations and demographic vulnerabilities, providing a visual representation of the systemic patterns observed in Table 2.

**Figure 11:**
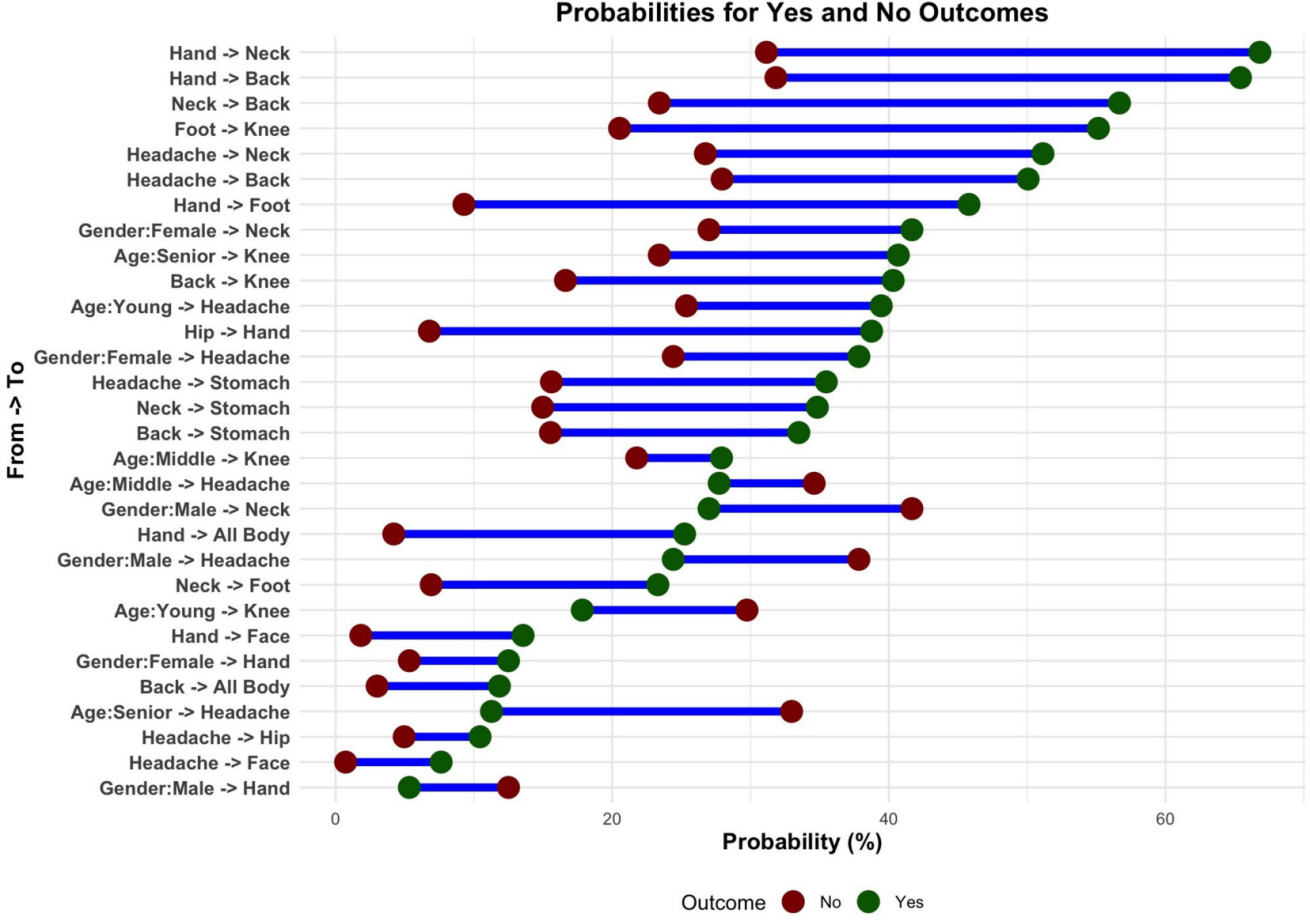
Probabilities of pain locations: green shows reported pain (“Yes”), red shows no pain (“No”), highlighting demographic trends and interconnections between pain locations.

Figure 11, also based on the data in Table 2, visualizes the probabilities of individuals reporting specific pain locations (“Yes”) vs. not reporting them (“No”) for different demographic and inter-symptom relationships. The horizontal bars represent the percentage probabilities, with green dots indicating positive responses (“Yes”) and red dots indicating negative responses (“No”). Key patterns emerge, such as the high probability of individuals with hand pain also reporting neck pain, back pain, or systemic pain (as seen in the larger green segments for these categories). Similarly, headache shows strong associations with neck pain, back pain, and stomach pain, reflecting interconnected pain locations.

## 4 Discussion

Our Bayesian network analysis revealed a complex web of probabilistic dependencies among chronic pain locations and demographic factors, underscoring the overlapping nature of pain syndromes and the need for targeted, personalized interventions. By quantifying these interconnections via odds ratios (ORs), we highlight key “hub” sites—particularly hand and facial pain—and demographic modifiers that may serve as early indicators of more widespread pain.

### 4.1 Gender-specific biopsychosocial pathways

We observed pronounced gender disparities across systemic pain conditions. Females represented 70% of those reporting generalized body pain and hand pain, and exhibited elevated odds for headache (OR = 1.88), neck pain (OR = 1.93), and hand pain (OR = 2.54). Beyond hormonal fluctuations (e.g., estrogen's modulation of nociceptive pathways), autoimmune disorders (which disproportionately affect women) and stress-related HPA-axis dysregulation likely contribute to these patterns Nijs et al. (14); Ji et al. (15). Central sensitization—a process of amplified CNS signaling—and nociplastic mechanisms further explain the co-occurrence of distinct pain sites in females, aligning with the chronic overlapping pain conditions framework Maixner et al. (16). Clinically, this suggests that women presenting with hand or facial pain warrant early assessment for systemic sensitization.

### 4.2 Key network hubs: hand and facial pain

Hand pain emerged as a potent trigger site, with ORs of 8.41 for facial pain, 8.25 for foot pain, and 7.68 for generalized pain. Its dense peripheral innervation and frequent overuse may amplify central nociceptive gain, precipitating multisite sensitization Tracey and Mantyh (17). Facial pain, in turn, was highly dependent on headache and hand pain (OR = 11.30), reflecting shared trigeminal and brainstem circuitry Cook and Chastain (18); Schwedt et al. (19). These hubs underscore strategic intervention targets: for example, early physiotherapy for hand pain or multimodal migraine management may disrupt downstream pain propagation.

### 4.3 Age-related shifts and demographic considerations

We confirmed an age-dependent transition from systemic to localized musculoskeletal pain. Younger adults reported higher rates of headache (39.4%) and abdominal pain (26.9%), likely linked to lifestyle stressors, screen use, and IBS Lovell and Ford (20). Middle-aged individuals exhibited peaks in back (50.2%), neck/shoulder (55.8%), and knee pain (56.8%), consistent with cumulative mechanical strain and early osteoarthritis Zhang and Jordan (21); Coté et al. (22). We deliberately defined “seniors” as ≥61 years (to preserve sufficient sample size), finding elevated knee (40.7%) and foot pain (16.2%), but markedly lower systemic pain. While this cutoff ensured robust estimates, it differs from the common ≥65 year threshold, which should be considered when comparing to other cohorts.

### 4.4 Clinical implications and personalized strategies

**Figure 12:**
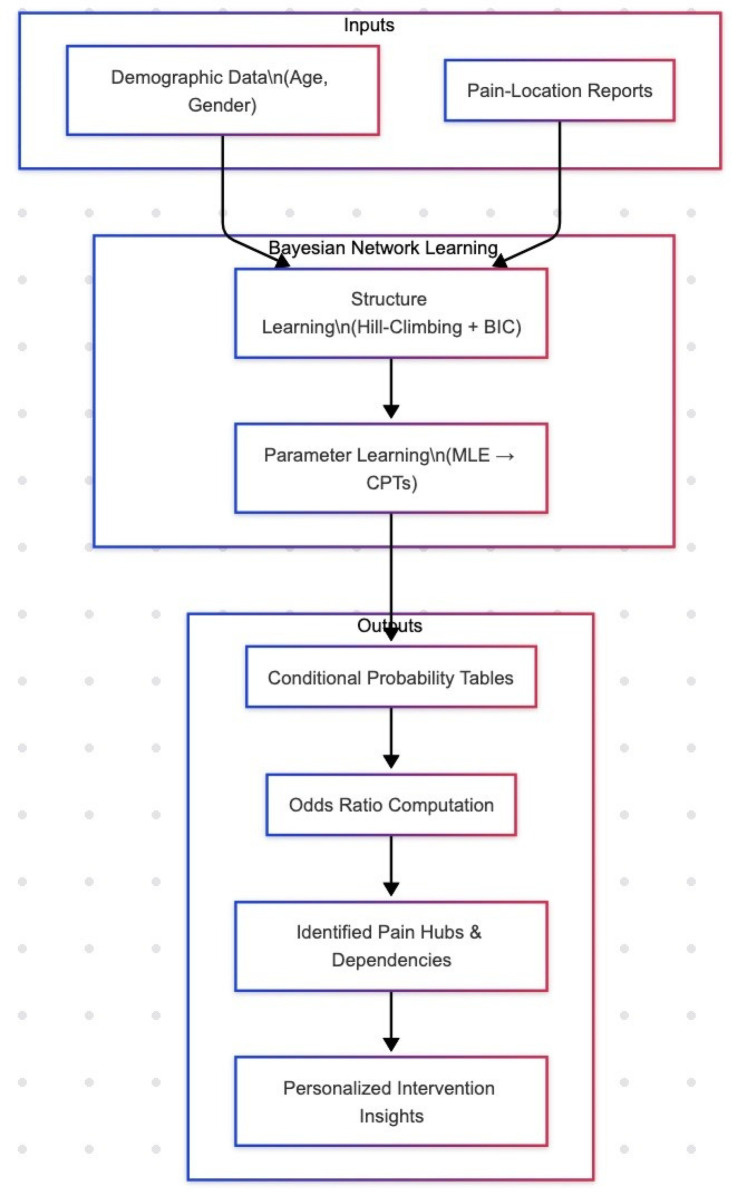
Analytic pipeline for Bayesian network modeling. We begin with *Inputs*—demographic covariates (age, gender) and self-reported pain-location data—fed into the *Structure Learning* phase (hill-climbing with BIC) to uncover the DAG of conditional dependencies. Next, *Parameter Learning* (MLE) estimates conditional probability tables (CPTs). From these, we compute *Odds Ratios* (ORs), which quantify the strength and direction of relationships (OR > 1 indicates increased likelihood; OR < 1 indicates decreased likelihood). The highest ORs identify *Pain Hubs & Dependencies* (e.g., hand pain, facial pain), which then inform *Personalized Intervention Insights* by highlighting key targets for early, tailored clinical strategies.

Our analytic pipeline (Figure 12)—from demographic and pain inputs through BN structure and parameter learning—yields conditional probabilities and ORs that pinpoint critical nodes for intervention. Targeted approaches, such as hormonal modulation in women with recurrent headaches or tailored hand rehabilitation, could preempt central sensitization and mitigate multisite pain. Embedding these probabilistic insights into clinical decision support tools may optimize resource allocation and patient outcomes. Our analytic pipeline (Figure 12)—from demographic and pain inputs through BN structure and parameter learning—yields conditional probabilities and ORs that pinpoint critical nodes for intervention. Targeted approaches, such as hormonal modulation in women with recurrent headaches or tailored hand rehabilitation, could preempt central sensitization and mitigate multisite pain. Embedding these probabilistic insights into clinical decision support tools may optimize resource allocation and patient outcomes.

### 4.5 Limitations and future directions

This study has several limitations:

1) Age cutoff and sample size. We defined “seniors” as ≥61 years to maintain adequate sample size; however, this differs from the more common ≥ 65 year standard and may affect comparability with other studies.
2) Self-report bias and lack of severity grading. Pain locations and duration (¿3 months) are self-reported without clinical validation or intensity scales, which may introduce recall and reporting biases.
3) Cross-sectional design and BN sensitivity. While BNs uncover conditional dependencies, they cannot establish causality or temporal directionality. Structure learning is data-driven and sensitive to sample size and variable discretization.
4) Generalizability. Our findings derive from a Qatari cohort and require replication in diverse populations.

Future research should integrate longitudinal data, biomarker and imaging measures, and quantitative sensory testing to refine network structures and validate risk patterns against treatment outcomes. Such efforts will advance precision pain medicine by linking mechanistic biomarkers with probabilistic modeling.

## 5 Conclusion

The results of this study provide a comprehensive understanding of chronic pain dynamics through a Bayesian Network (BN) model. While barplot visualizations initially suggested that all pain variables were uniformly linked to the gender variable, the BN analysis revealed a more intricate interplay. Specifically, gender influenced certain pain variables such as neck or shoulder pain and headache or migraine, showing stronger associations in females compared to males, particularly when combined with hand pain. These findings underscore the sophistication of BN analysis, which allows for a deeper exploration of conditional dependencies and interactions.

Moreover, the study sheds light on the significant role of demographic factors like age and gender in chronic pain pathways. For instance, the model demonstrated that younger individuals had a higher likelihood of reporting headaches compared to older cohorts, with seniors showing markedly reduced probabilities. Similarly, the analysis revealed that females are disproportionately affected by certain pain locations, such as neck or shoulder pain, a trend that is amplified in the presence of co-occurring conditions like hand pain or headaches. These insights emphasize the importance of tailoring pain management strategies to demographic characteristics, ensuring interventions are both gender-sensitive and age-appropriate.

Additionally, the BN model illustrated how specific pain locations, such as back pain, act as central nodes influencing the broader pain network. For example, back pain showed strong conditional dependencies with both neck pain and widespread body pain, highlighting the interconnectedness of musculoskeletal and systemic pain syndromes. This underscores the necessity of addressing primary pain sources to mitigate cascading effects throughout the pain network.

By leveraging the BN framework, this study provides a robust methodological approach for disentangling complex relationships in chronic pain. It offers actionable insights into how demographic factors and localized pain locations interact to propagate or mitigate overall pain experiences. The model's ability to elucidate these intricate pathways can guide the development of more effective, personalized interventions aimed at reducing the burden of chronic pain. This nuanced approach offers a promising framework for advancing both the understanding and management of chronic pain.

## Data Availability

The raw data supporting the conclusions of this article will be made available by the authors, without undue reservation.
